# GSDMD-mediated pyroptosis dominantly promotes left ventricular remodeling and dysfunction in post-myocardial infarction: a comparison across modes of programmed cell death and mitochondrial involvement

**DOI:** 10.1186/s12967-023-03873-6

**Published:** 2023-01-10

**Authors:** Chanon Piamsiri, Chayodom Maneechote, Kewarin Jinawong, Busarin Arunsak, Titikorn Chunchai, Wichwara Nawara, Siriporn C Chattipakorn, Nipon Chattipakorn

**Affiliations:** 1 Cardiac Electrophysiology Research and Training Center, Faculty of Medicine, Chiang Mai University, Chiang Mai, 50200 Thailand; 2 Cardiac Electrophysiology Unit, Department of Physiology, Faculty of Medicine, Chiang Mai University, Chiang Mai, 50200 Thailand; 3 Center of Excellence in Cardiac Electrophysiology Research, Chiang Mai University, Chiang Mai, 50200 Thailand; 4 Department of Oral Biology and Diagnostic Sciences, Faculty of Dentistry, Chiang Mai University, Chiang Mai, 50200 Thailand

**Keywords:** Myocardial infarction, Heart failure, Programmed cell death, Pyroptosis, Mitochondria

## Abstract

**Background:**

Myocardial infarction (MI) has recently accounted for more than one-third of global mortality. Multiple molecular pathological pathways, such as oxidative stress, inflammation, and mitochondrial dysfunction, have been recognized as possible mechanisms in the development of MI. Furthermore, different phases of ischemic injury following the progression of MI were also associated with multiple types of programmed cell death (PCDs), including apoptosis, necroptosis, ferroptosis, and pyroptosis. However, it remains unknown whether which types of PCDs play the most dominant role in post-myocardial infarction (post-MI).

**Method:**

In this study, we used a preclinical rat model of MI induced by permanent left anterior descending coronary artery (LAD) ligation (n = 6) or a sham operated rat model (n = 6). After a 5-week experiment, cardiac function and morphology, mitochondrial studies, and molecular signaling analysis of PCDs were determined.

**Results:**

Herein, we demonstrated that post-MI rats had considerably impaired cardiac geometry, increased oxidative stress, myocardial injuries, and subsequently contractile dysfunction. They also exhibited worsened cardiac mitochondrial function and dynamic imbalance. More importantly, we found that post-MI mediated abundant myocardial cell death through multiple PCDs, including apoptosis, necroptosis, and pyroptosis, but not ferroptosis.

**Conclusion:**

In this study, we provide the first insights into the mechanism of PCDs by pyroptosis, which is leveraged as the most dominant mode of cell death after MI.

## Background

Cardiovascular disease has recently been reported as being the leading cause of death globally, accounting for 110 million cases and approximately 19 million deaths globally [1]. Of these deaths, myocardial infarction (MI) is the most important cause of cardiovascular disease-related death [1, 2]. MI is defined as the death of myocardial cells attributable to the interruption of coronary perfusion [3, 4]. Myocardial cell death is known to be a hallmark characteristic of many cardiovascular diseases including acute MI, myocardial ischemia/reperfusion injury (I/R), and heart failure (HF) [5, 6]. Recently, it has been observed that several forms of programmed cell death (PCD), including apoptosis, necroptosis, ferroptosis, and pyroptosis, play separate roles during the pathological progression of various cardiac diseases, particularly post-MI [6–12]. However, current knowledge regarding the contribution of these PCDs after MI is largely based on very limited information. Although different types of cell death including apoptosis, pyroptosis, necroptosis and ferroptosis have been reported, they were not simultaneously investigated in the same study model and thus could not provide important insights whether which type of cell death plays a dominant role in the setting of post-MI injury. Thus, understanding the molecular phenotype of the PCDs underlying the pathophysiological processes of MI may facilitate the development of better therapeutic approaches for MI patients.

Mitochondrial dysfunction and impaired mitochondrial dynamic balance are known to be related to impaired cellular energy regulation and excessive oxidative stress, which also contribute to the deterioration of myocardial function in HF [13, 14]. Excessive mitochondrial fission and decreased mitochondrial fusion have been shown to increase mitochondrial damage and fragmentation, resulting in increased severity of HF [15–19]. However, the changes involved in mitochondrial function and mitochondrial dynamic control in post-MI pathology have not been widely investigated.

In this study, our aims were: (1) to characterize the dominant form of programmed cell death, and (2) to investigate the role of mitochondrial dynamics in the rat models after MI. We hypothesized that post-MI the mitochondrial dynamic balance is impaired and involves multiple PCDs including apoptosis, necroptosis, ferroptosis, and pyroptosis. Findings from this study would provide valuable insights and confer a potential precise cardioprotective treatment for the post-MI patients.

## Methods

### Animal preparation

All male Wistar rats (350–400 g body weight; Nomura Siam International Co., Ltd, Bangkok, Thailand) were used. Since previous studies reported that female gonadal hormones were associated with cardioprotection, most researchers avoid using female animals since their variable nature may introduce experimental variability [20]. In accordance with the unsteady nature of female data brought on by hormonal variations linked with the female reproductive cycle, only male rats were included in this study. All experimental procedures were approved by the Laboratory Animal Center (Office of Research Administration, Chiang Mai University) and the Chiang Mai University Animal Care and Use Committee, Chiang Mai University (permit number 2564/RT0001) and were carried out in accordance with the recommendations of the Guide for the Care and Use of Laboratory Animals.

### Animal model of MI

**Fig. 1:**
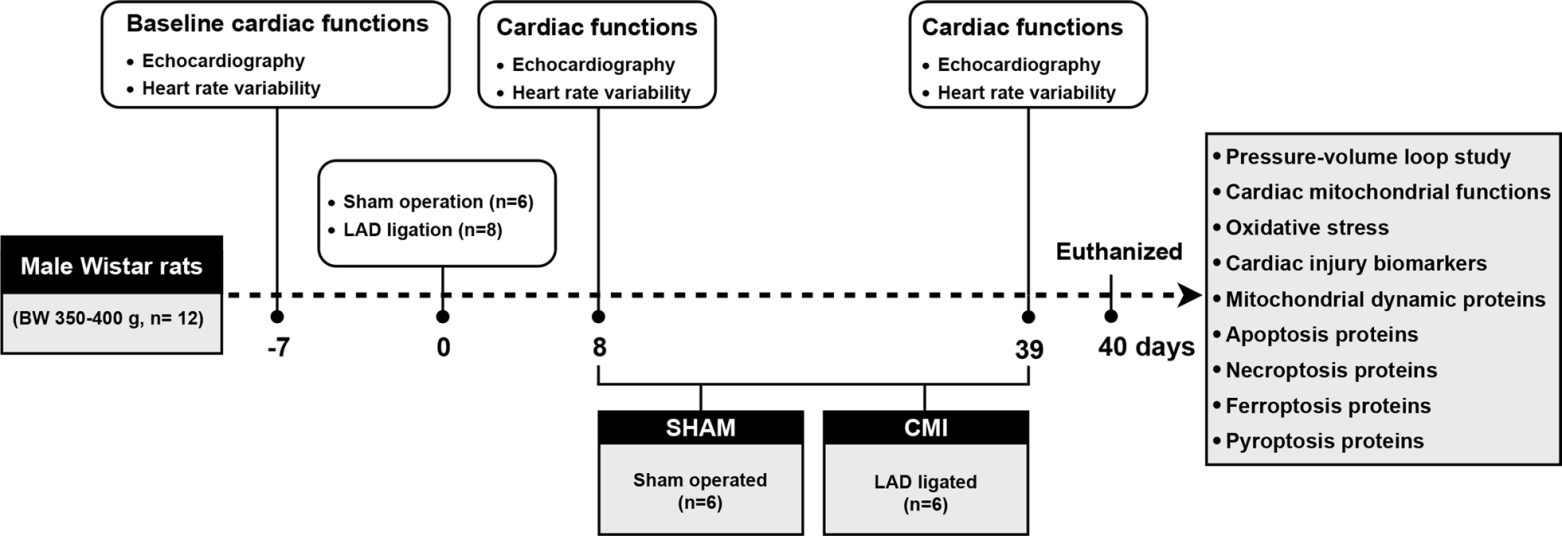
Schematic diagram of the experimental design, n = 8 for the MI group and n = 6 for the Sham group. For the MI group, two rats were excluded, one rat died within 24 h after the surgery and one rat did not develop anterior wall akinesis. Eight days after the surgery, all rats (SHAM, n = 6; MI, n = 6) were sacrificed on day 40 for cardiac tissue and biomolecular studies

After allowing for acclimatization of the rats, a left thoracotomy was carried out under general anesthesia and the LAD coronary artery was identified and permanently occluded with a sterile 5−0 suture approximately 2 to 3 mm from the atrial appendage. Visible paling and cyanotic discoloration of the anterior wall of the left ventricle (LV) were taken as indicative of successful ligation. Rats that underwent left thoracotomy without LAD ligation served as the sham control group. Transthoracic echocardiography was performed on day 8 after surgery to assess cardiac morphology and function and to confirm the development of the MI model. The rats that did not have anterior wall akinesis and cardiac fibrosis were excluded from the study. On day 40, all animals were euthanized for cardiac tissue and biomolecular studies. The experimental design of this study was shown in Fig. 1.

### Cardiac function assessment

Noninvasive cardiac function parameters were determined by transthoracic echocardiography (Philips Affiniti 50 C, Amsterdam, Netherlands). Parasternal short-axis and M-mode echocardiography were performed at the midventricular level. The LV systolic parameters, including a % LV ejection fraction (%LVEF) were evaluated. The transmitral E-wave/A-wave (E/A) velocity ratio was recorded from the apical four-chamber view to represent LV diastolic function.

Other cardiac functions were determined invasively by a LV pressure-volume (P-V) loop study at the end of the study period. In brief, general anesthesia was induced using Xylazine HCl (LBS laboratory ltd. Part, Bangkok, Thailand), 5 mg/kg in combination with 50 mg/kg Zoletil® (Virbac Laboratories, Carros, France) via intramuscular injection. The P-V catheter (Transonic Scisense, Ontario, Canada) was cannulated and advanced through the right carotid artery into the LV to determine pressure and volume. Invasive cardiac function parameters including heart rate (HR), LV end-systolic pressure (LVESP), LV end-diastolic pressure (LVEDP), LV end-diastolic volume (LVEDV), and LV end-systolic volume (LVESV), cardiac output (CO), and stroke volume/body weight (SV) were observed.

### Heart rate variability (HRV) study

Heart rate variability (HRV) data were obtained from an electrocardiogram with standard limb lead II (PowerLab 4/25T, AD Instrument, Sydney, Australia). The high-frequency (HF) component (0.15–0.40 Hz) was considered as being a marker of parasympathetic activity. The association between sympathetic and parasympathetic activity was regarded as low-frequency (LF; 0.2–0.6 Hz). LF/HF ratio was considered as an index for cardiac sympathetic and parasympathetic tone. An increasing LF/HF ratio indicated an increase in sympathetic and/or a decrease in parasympathetic tone [21].

### Determination of cardiac mitochondrial membrane potential (ΔΨm), reactive oxygen species (mtROS) and swelling

Freshly-isolated mitochondria were stained with either 5,5′,6,6′-tetrachloro-1,1′,3,3′-tetraethylbenzimidazolecarbocyanine iodide (JC-1, Sigma-Aldrich, Missouri, USA) or dichlorohydrofluorescein diacetate (DCFDA, Sigma-Aldrich, Missouri, USA) for the determination of ΔΨm (485 nm) or mtROS (485 nm) using a spectrophotometer (Gen5 Microplate Reader, BioTek Instruments, Vermont, USA) [22–24]. In addition, cardiac mitochondrial swelling was also determined by measuring the change in absorbance density under a spectrophotometer. The decrease in absorbance of the suspension at 540 nm indicates mitochondrial swelling [23].

### Cardiac injury biomarkers and oxidative stress (cTnI, NT-proBNP, and MDA quantification)

Biomarkers of cardiac injury, including the levels of cardiac troponin-I (cTnI) and N-terminal pro B-type natriuretic peptide (NT-proBNP) (MyBioSource, Inc., San Diego, USA), were determined using ELISA assay kits in accordance with the manufacturer’s instructions [23]. For oxidative stress, the levels of malondialdehyde (MDA) concentrations in serum and cardiac tissue were determined using the thiobarbituric acid method and measured with the high-performance liquid chromatography (HPLC) system as previously described [23, 25].

### Western blot analysis

The proteins used for western blotting were harvested from the fresh myocardium at the end of the study period and separated by sodium dodecyl sulfate-polyacrylamide gel electrophoresis (SDS-PAGE). The membranes were immunoblotted with antibodies against DRP1, pDRP1, MFN1, MFN2, OPA1, Cytochrome C, Bax, Bcl-2, Caspase 3, Cleaved-caspase 3, RIPK3, pRIPK3, MLKL, pMLKL, NLRP3, ACSL4, GPX4, GSDMD, GSDMD-NT, and GAPDH overnight. Subsequently, anti-mouse IgG conjugated with horseradish peroxidase was applied. Enhanced chemiluminescence (ECL) detection reagents were administered to visualize peroxidase reaction products. The immunoreactive bands were visualized under a chemiluminescence imaging system (Bio-Rad Laboratories, USA). The band density was quantified using ImageJ analysis.

### Statistical analysis

All data were expressed as mean ± standard error of the mean (mean ± SEM). To determine differences between groups, a Student’s t-test or a one-way analysis of variance (ANOVA) with Fisher’s LSD post hoc test was performed. The normality of the dataset was tested using the Shapiro-Wilk test. Statistical significance was considered at *p* < 0.05.

## Results

### Post-MI mediated cardiac dysfunction and heart failure

**Fig. 2:**
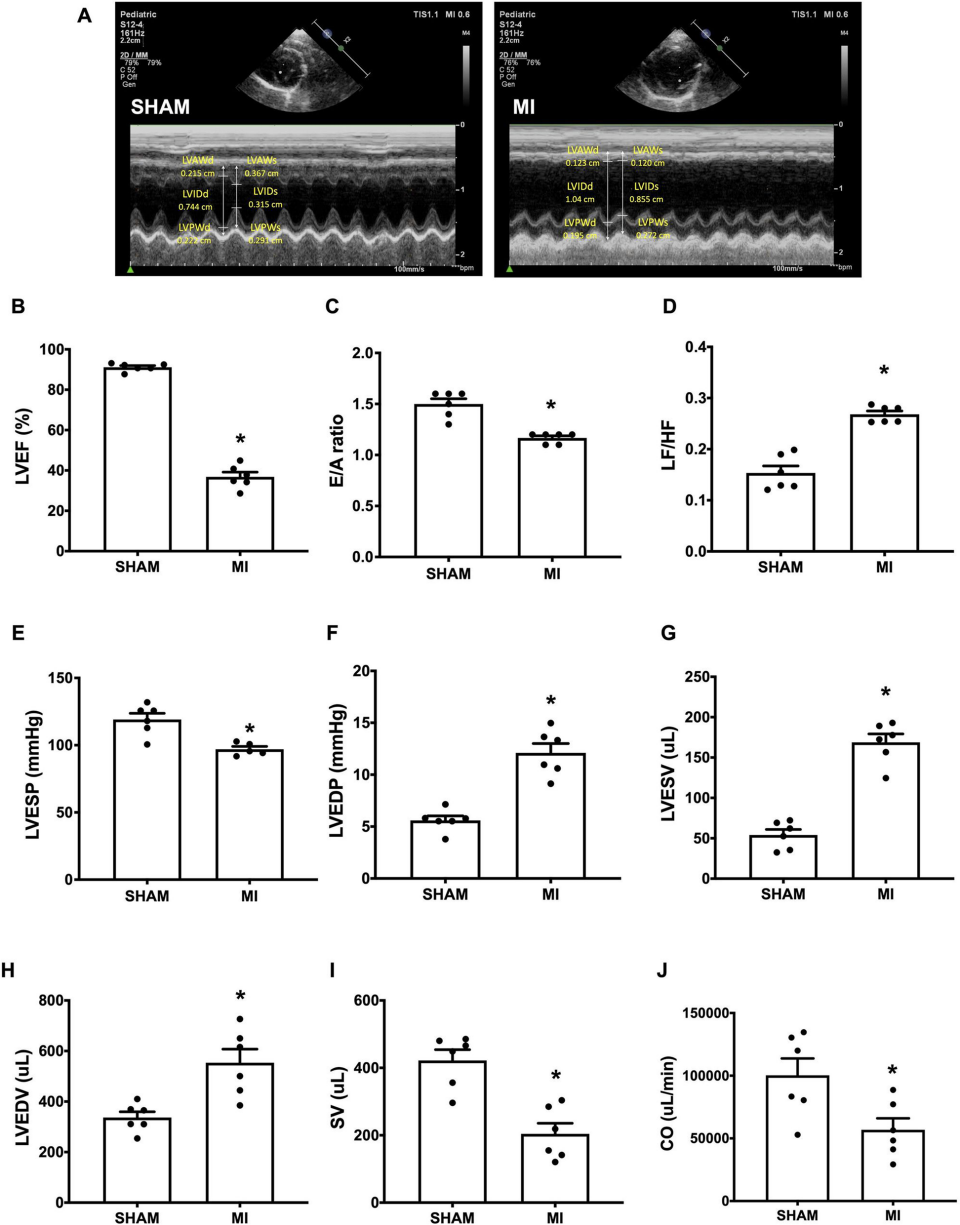
Post-MI mediated cardiac dysfunction and HF. **A** representative images of transthoracic echocardiography of SHAM and post-MI rats at 5-weeks; **B** the quantitative analysis of LVEF; **C** E/A ratio; **D** LF/HF; **E** LVESP; **F** LVEDP; **G** LVESV; **H** LVEDV; **I** SV; **J** CO; n = 6 per group. All bar charts are expressed as mean ± SEM. *p < 0.05 vs. SHAM. AI, myocardial infarction; LVEF, left ventricular ejection fraction; E/A ratio, early passive diastolic filling to active diastolic filling velocities ratio; LF/HF, low frequency to high frequency ratio; LVESP, left ventricular end-systolic pressure; LVEDP, left ventricular end-diastolic pressure; LVESV, left ventricular end-systolic volume; LVEDV, left ventricular end diastolic volume; SV, stroke volume; CO, cardiac output

We observed that one rat died within 24 h after LAD occlusion and one rat did not have anterior wall akinesis during the echocardiography confirmation eight days following surgery. In the sham-operated group, there was no mortality. The representative echocardiography images revealed that post-MI rats developed LV remodeling as evidenced by thinning of both the LV anterior wall (LVAW) and LV posterior wall (LVPW) during systole and diastole, in comparison with the sham-operated rats (Fig. 2A). LV wall motion abnormalities with anterior wall akinesia were observed in the post-MI rats when compared to the sham rats (Fig. 2A). Post-MI rats also exhibited significantly impaired LV systolic function, as indicated by a 61% reduction in LVEF compared with the sham rats (Fig. 2B). A significant decrease in the ratio of trans-mitral flow during early to late diastolic filling velocity (E/A) was found in the post-MI rats in comparison with the sham rats, indicating a dramatic impairment of LV diastolic function following the 5-week LAD occlusion (Fig. 2C). We also found that post-MI rats had an increased LF/HF ratio which indicated cardiac sympathovagal imbalance after MI. In addition to echocardiography, invasive P-V loop analysis was also performed in this study. Post-MI hearts had increased LVEDV and LVESV, but had decreased SV and CO, when compared with sham hearts, indicating LV-volume overload and systolic dysfunction (Fig. 2G**–**J).

## Post-MI mediated myocardial injuries and HF

**Fig. 3:**
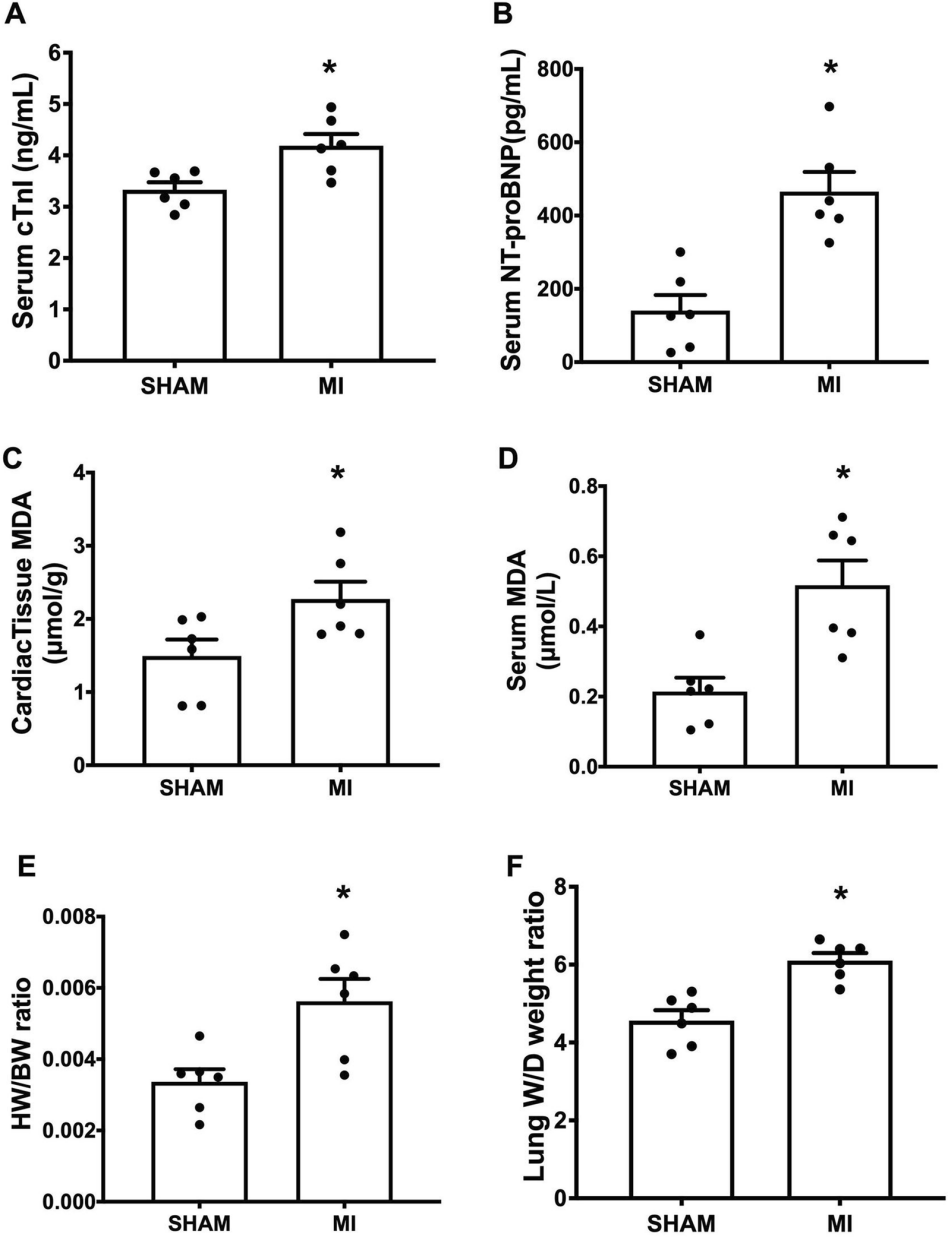
Post-MI mediated myocardial injury, oxidative stress and HF. Quantitative analysis of: **A** Serum cTnI levels; **B** Serum NT-proBNP levels; **C** Cardiac tissue MDA levels; **D** Serum MDA levels; **E** HW/BW ratio; **F** Lung W/D weight ratio; n = 6 per group. All bar charts are expressed as mean ± SEM. *p < 0.05 vs. SHAM. MI, myocardial infarction; cTnI, cardiac troponin I; NT-proBNP, N-terminal pro B-type natriuretic peptide; MDA, malondialdehyde; HW/BW ratio, heart weight to body weight ratio; Lung W/D weight ratio, lung wet-to-dry weight ratio

To determine whether myocardial injury was mediated post-MI, the markers cTnI and NT-proBNP were measured. Serum levels of both cTnI and NT-proBNP were significantly increased in the MI group, compared to the sham group (Fig. 3A, B). In addition, cardiac tissue and serum MDA levels were also increased in the MI group when compared to the sham group, indicating the presence of myocardial injury and oxidative damage after MI (Fig. 3C, D). The heart to body weight ratio (HW/BW) and wet to dry lung weight ratio (Lung W/D) were measured, and the results showed that MI rats had significantly higher values for HW/BW and Lung W/D in comparison to sham rats, indicating cardiac hypertrophy and congestive HF, respectively (Fig. 3E, F). Collectively, 5-week post-MI resulted in myocardial injury, cardiac dysfunction, and eventually HF.

## Post-MI mediated cardiac mitochondrial dysfunction and mitochondrial dynamic imbalance

**Fig. 4:**
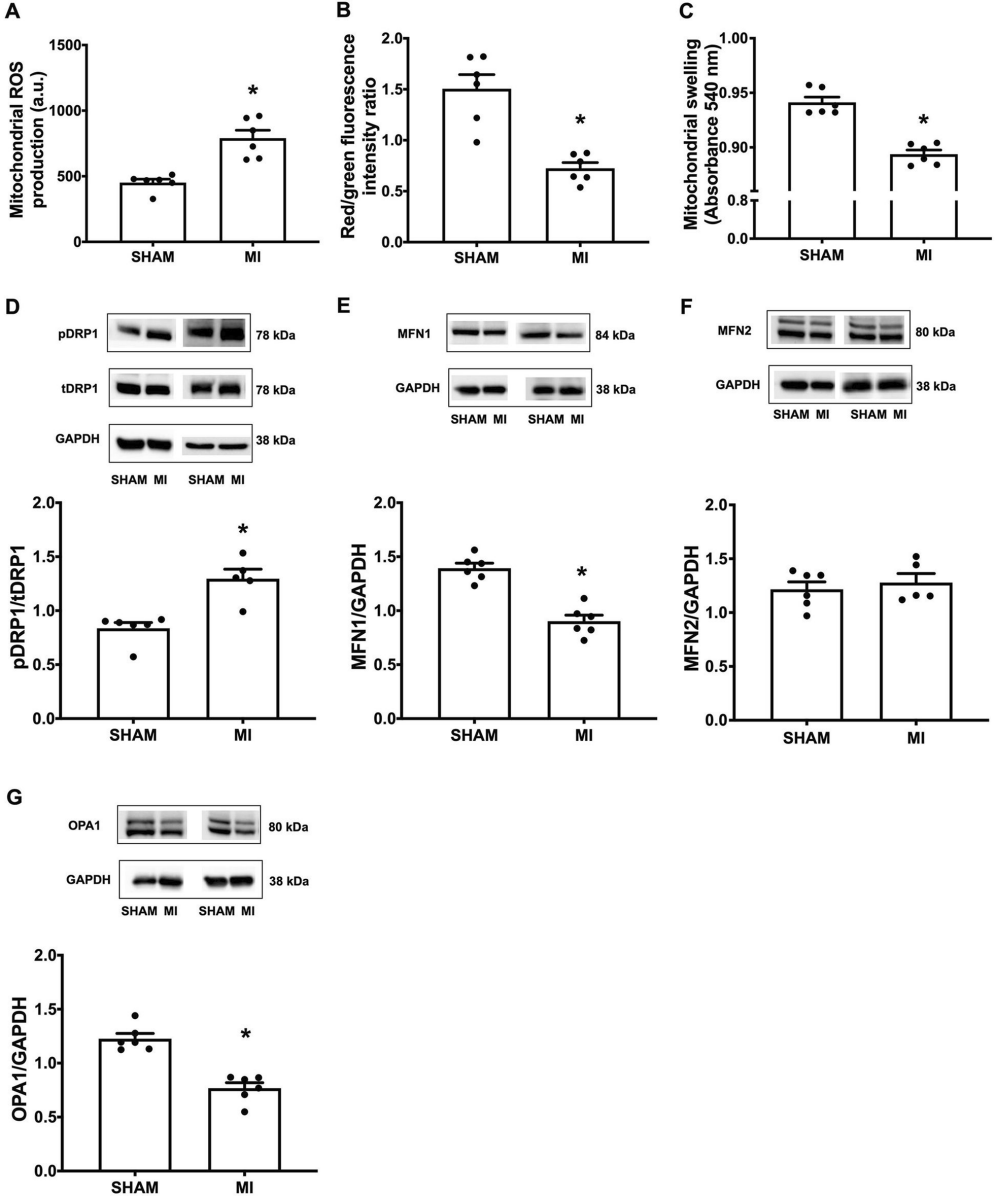
Post-MI mediated cardiac mitochondrial ROS over-production, membrane depolarization and mitochondrial swelling. Post-MI increased mitochondrial fission and reduced mitochondrial fusion. Quantitative analysis of: **A** Mitochondrial ROS production; **B** Mitochondrial membrane potential; **C** Mitochondrial swelling; **D** cardiac tissue pDRP1/tDRP1; **E** cardiac tissue MFN1/GAPDH; **F** cardiac tissue MFN2/GAPDH; **G** cardiac tissue OPA1/GAPDH; n = 5–6 per group. All bar charts are expressed as mean ± SEM. *p < 0.05 vs. SHAM. *MI* myocardial infarction; *ROS* reactive oxygen species

To date, although mitochondrial dysfunction and dynamic imbalance in acute MI and cardiac I/R have been extensively studied, details regarding these impairments remain elusive. In this study, we found that post-MI cardiac mitochondrial dysfunction occurred as indicated by excessive ROS production, mitochondrial depolarization (i.e. reduced red/green fluorescence intensity ratio), and mitochondrial swelling (i.e. reduced absorbance) when compared with the sham group (Fig. 4 A**–**C). Changes in cardiac mitochondrial dynamics has been shown to be one of the promising mechanistic strategies during the progression of various cardiac diseases. Therefore, we determined the expression of mitochondrial dynamic proteins, including pDRP1/tDRP1, MFN1, MFN2 and OPA1. Our results showed that the expression of mitochondrial fission proteins (pDRP1/tDRP1) was markedly increased in the MI group, along with a decrease in mitochondrial fusion proteins (i.e., MFN1 and OPA1), compared with the sham group (Fig. 3D and G). These findings indicated that impaired mitochondrial dynamic balance and deterioration of mitochondrial integrity and function contributed to LV dysfunction post-MI.

## Pyroptosis as the dominant form of PCD in post-MI hearts

**Fig. 5:**
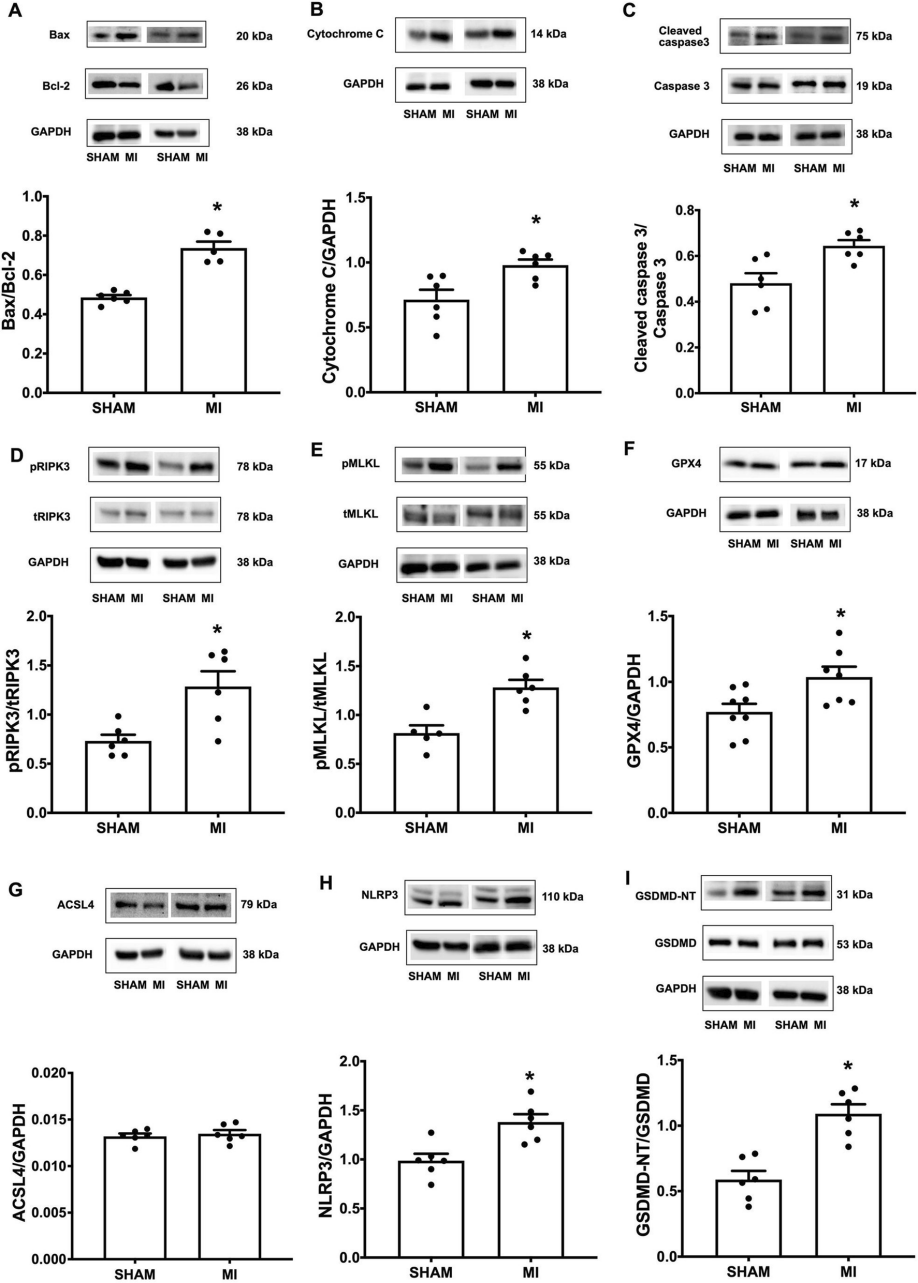
Post-MI mediated apoptosis, necroptosis and pyroptosis but not ferroptois. Quantitative analysis of: **A** Bax/Bcl-2; **B** Cytochrome C/GAPDH; **C** Cleaved-caspase 3/Caspase 3; **D** pRIPK3/tRIPK3; **E** pMLKL/tMLKL; **F** GPX4/GAPDH; **G** ACSL4/GAPDH; **H** NLRP3/GAPDH; **I** GSDMD-NT/GSDMD; n = 5–6 per group. All bar charts are expressed as mean ± SEM. *p < 0.05 vs. SHAM. *MI* myocardial infarction

To support the evidence that a chronic ischemic insult could lead to myocardial death, contractile dysfunction, and inevitable HF, we determined the pertinent proteins of each type of programmed cell death pathway, including Bax, Bcl-2, Cytochrome C, Caspase 3, Cleaved-caspase 3, RIPK3, pRIPK3, MLKL, pMLKL, GPX4, ACSL4, NLRP3, GSDMD, and GSDMD-NT. Our results indicated that the hallmark biomarkers of apoptosis (Bax/Bcl2, Cytochrome C and Cleaved-caspase 3/Caspase 3) (Fig. 5A–C), necroptosis (pRIPK3/tRIPK3, pMLKL/tMLKL) (Fig. 5D, E), and pyroptosis (NLRP3, GSDMD-NT/GSDMD) (Fig. 5H, I), but not ferroptosis (ACSL4) (Fig. 5G), were markedly increased in the myocardial tissue of the MI group in comparison with those of the sham group (Fig. 5). Altogether, we showed unique molecular findings indicating that post-MI mediated forms of PCD included apoptosis, necroptosis, and pyroptosis, but not ferroptosis.

**Fig. 6:**
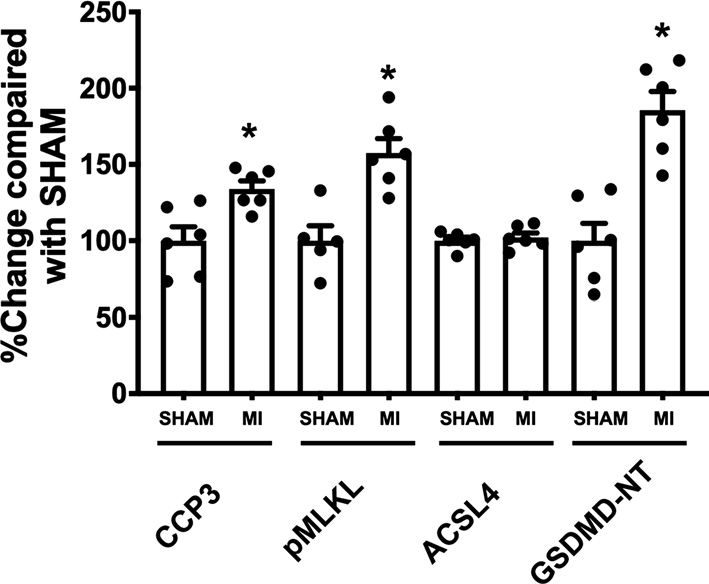
Contribution of PCDs post-MI. Relative change of executioner protein expression of each PCD: Cleaved-caspase 3 for apoptosis; pMLKL for necroptosis; ACSL4 for ferroptosis; GSDMD-NT for pyroptosis; n = 5–6 per group. All bar charts are expressed as mean ± SEM. *p < 0.05 vs. SHAM. Abbreviations: *MI*, myocardial infarction

Considering the changes of an executioner protein of each PCD (i.e., Cleaved-caspase 3 for apoptosis, pMLKL for necroptosis, ACSL4 for ferroptosis, and GSDMD-NT for pyroptosis) in the MI group compared to sham rats (Fig. 6), the %change in the expression levels of GSDMD-NT was the most significantly expressed protein in the cardiac tissue of post-MI rats (by 34, 57, 2 and 86% for Cleaved-caspase 3, pMLKL, ACSL4, and GSDMD-NT, respectively). These results suggested that pyroptosis was the dominant PCD involved post-MI.

## Discussion

Ischemic heart disease is responsible for more than one-third of worldwide mortality [1, 5]. MI has been ranked as one of the most important ischemic pathologies which causes extensive cardiomyocyte death, leading to LV remodeling and HF [2, 4, 26]. As a result, comprehensive studies of potential molecular signaling and associated mechanisms during post-MI progression would be beneficial in informing the development of new therapeutic strategies and therapies in the clinical setting of post-MI patients. In this study, we performed permanent LAD ligation to mimic MI pathology in rats. The main outcomes are as follows: (i) Post-MI mediated myocardial injury, cardiac systolic and diastolic dysfunction, and subsequently HF; (ii) Post-MI impaired cardiac mitochondrial dynamic balance by increasing mitochondrial fission but decreasing mitochondrial fusion, which in turn impaired mitochondrial function; (iii) Post-MI mediated several PCDs, including apoptosis, necroptosis, and pyroptosis, but not ferroptosis; (iv) pyroptosis was the most dominant form of PCD in post-MI pathology.

HF is acknowledged as being the fatal consequence in about half of all patients with an unfavorable prognosis of MI [27, 28]. HF could develop soon after acute MI or gradually post-MI [29]. In the current study, our results confirmed that post-MI there were adverse biomechanical changes in the heart, resulting in impaired cardiac contractile function in both systolic and diastolic phases. Similarly, post-MI-induced cardiac geometry changed by the induction of LV structural thinning and dilatation. As a consequence of sustained ischemic insults, myocardial injury and cardiomyocyte death occurred, as indicated by elevated levels of NT-proBNP and cTnI in peripheral blood, which were associated with LV wall stress and myocardial cell injury. An increase in myocardial injury post-MI has also been reported to be associated with an increase in oxidative stress, inflammation, and mitochondrial dysfunction, resulting in cell death in the ischemic myocardium [30, 31].

Mitochondrial function is governed by a morphologically controlled mechanism of mitochondria, known as ‘mitochondrial dynamics’ in which fission and fusion processes are coordinated [17, 32]. These phenomena ensure the appropriate balance of energy demand and supply in the mitochondria. Previous studies have reported that excessive mitochondrial fission and decreased mitochondrial fusion increased the severity of HF [15–19]. Consistent with these studies, we observed a significant increase in the fission markers (pDRP1/tDRP1 ratio) but a decrease in the fusion (MFN1 and OPA1) markers in post-MI rats, indicating a dynamic imbalance of mitochondria after MI. This finding was consistent with a previous report which demonstrated that OPA1 required MFN1, but not MFN2, to regulate the fusion process [33].

To date, the manifestations and mechanisms of various PCDs, including apoptosis, necroptosis, ferroptosis, and pyroptosis, have been identified as being associated with cardiovascular diseases [6–9, 11, 12]. Previous studies have shown that apoptosis was upregulated in the first 2 h and persisted up to 12 weeks after MI [9, 11, 26]. Wang X. and colleagues (2018) found that upregulation of apoptotic signaling, including Cleaved-caspase 3 and TUNEL positive cardiomyocytes, was detected 2-weeks after MI and remained elevated for weeks in the mouse model of post-MI [11]. In our study, we found a significant increase in apoptosis-related proteins, including Cytochrome C, Bax, and Cleaved-caspase3 in rats 5-weeks after MI. It is well known that apoptosis is responsible for early cardiomyocyte death and would trigger macrophage clearance but is less likely to induce inflammation [4]. In contrast to apoptosis, necroptosis is considered as a potent inflammatory inducer [34]. It has been shown that myocardial necroptosis was detected 1 to 12 weeks after MI in the mouse model of post-MI, as indicated by increased tRIPK1, tRIPK3, and tMLKL [12]. Consistently, we observed a marked increase in pRIPK3 and pMLKL protein levels, suggesting activation of necroptosis.

Ferroptosis is a novel iron-dependent form of PCD characterized by the accumulation of ROS, leading to lipid peroxidation of polyunsaturated fatty acids in plasma membranes, resulting in membrane breakdown [6, 7, 35]. Glutathione peroxidase 4 (GPX4) serves as an endogenous regulator of lipid peroxidation. The depletion of GPX4 would lead to lipid peroxidation and ferroptosis [7, 35]. It has been reported that acute oxidative injury exacerbated myocardial ferroptosis in SD rats after 1-week MI [36]. In our study, the MI rats had significantly higher MDA levels in both cardiac tissue and serum than sham rats, which indicated lipid peroxidation. Surprisingly, we also found that the expression of GPX4 levels were increased in MI rats, whereas no significant change in the expression of ACSL4 was found. These findings suggested that ferroptosis was declining in 5-weeks MI. This is confirmed by a previous report demonstrating that GPX4 levels were decreased in the acute and middle stages of MI (i.e., 1 day to 1 week after LAD ligation), but subsequently increased in the late phase (i.e. 8 weeks of MI) [10]. Taken together, all of these findings suggested that ferroptosis was declining due to the compensatory increased antioxidant regulation of GPX4 after MI.

Pyroptosis is known as an inflammation mediated cell death as a result of the priming and activating of the inflammasome, which subsequently increases the permeability of the plasma membrane and releases inflammatory cytokines [6]. Inflammasome activation and pyroptotic cell death have been identified in various cardiovascular disease models, including acute MI, cardiac I/R, and HF [8, 37–39]. Prior to this report, the current understanding of the molecular phenotype of the dominant PCDs involved in post-MI was still elusive.

Our findings revealed for the first time that the pyroptosis executor protein GSDMD-NT was also upregulated in the post-MI model. In summary, we demonstrated that chronic progression of MI mediates multiple PCDs, including apoptosis, necroptosis, and pyroptosis, but not ferroptosis. And pyroptosis was the most dominant form of PCD in post-MI pathology.

This study provided the first insights on a significant escalation of pyroptosis activation as a candidate therapeutic target for post-MI, together with the demonstration of the mechanistic insights across different modes of programmed cell death and mitochondrial involvement. Our findings highlight a potential future therapeutic opportunity for pyroptosis, which could represent a novel therapeutic approach for post-MI patients. However, our preliminary findings used a small sample size. Also, the potential benefits of specific inhibition of each PCDs in post-MI pathology were not investigated in this study. Hence, future studies using various inhibitors of PCDs are needed to warrant its translation to a clinical setting of post-MI.

## Conclusion

In conclusion, our findings revealed that pyroptosis was the most dominant form of PCD in post-MI pathology. Our findings also suggest potential future clinical translation targeting pyroptosis as a novel cardioprotective approach for post-MI.

## Data Availability

Not applicable.

## Abbreviations

ACSL4: acyl-CoA synthetase long chain family member 4
Bax: bcl-2-like protein 4
Bcl-2: B-cell lymphoma 2
CAD: Coronary artery disease
CO: Cardiac output
cTnI: Cardiac troponin I
DRP1: Dynamin-related protein 1
E/A: Trans-mitral flow during early to late diastolic filling velocity
GPX4: Glutathione peroxidase 4
GSDMD: Gasdermin D
GSDMD-NT: Gasdermin D N terminal
HF: Heart failure
HW/BW: Heart weight to body weight
I/R: Ischemia reperfusion injury
LAD: Left anterior descending coronary artery
LV: Left ventricle
LVAW: Left ventricular anterior wall
LVPW: Left ventricular posterior wall
Lung W/D: Lung wet to dry weight
MI: Myocardial infarction
MFN1: Mitofusin 1
MFN2: Mitofusin 2
MLKL: Mixed lineage kinase domain like pseudokinase
NLRP3: NLR family pyrin domain containing 3
NT-proBNP: N-terminal pro b-type natriuretic peptide
OPA1: Optic atrophy 1
PCD: Programmed cell death
pDRP1: Dynamin-related protein 1 (phosphorylated)
pRIPK1: Receptor-interacting serine/threonine-protein kinase 1 (phosphorylated)
pRIPK3: Receptor-interacting serine/threonine-protein kinase 3 (phosphorylated)
RIPK1: Receptor-interacting serine/threonine-protein kinase 1
RIPK3: Receptor-interacting serine/threonine-protein kinase 3
ROS: Reactive oxygen species
SV: Stroke volume
tMLKL: Mixed lineage kinase domain like pseudokinase (total form)
TUNEL: Terminal deoxynucleotidyl transferase-mediated dUTP nick end labeling
